# Effects of Rice Husk Ash Particle Size and Luxan Value Influence on Mortar Properties and Proposal of Hydration Ratio Measurement Method

**DOI:** 10.3390/ma18010021

**Published:** 2024-12-25

**Authors:** Junho Kim, Hikaru Fumino, Manabu Kanematsu

**Affiliations:** 1 Department of Architecture, Faculty of Science and Technology, Tokyo University of Science, Noda City 278-8510, Japan; manabu@rs.tus.ac.jp; 2 National Institute of Technology (KOSEN), Oyama College, Oyama City 323-0806, Japan; fumino@oyama-ct.ac.jp

**Keywords:** rice husk ash, pozzolanic material, particle size, hydration ratio

## Abstract

A fundamental study has been conducted on the effective utilization of rice husk ash (RHA) in concrete. RHA is an agricultural byproduct characterized by silicon dioxide as its main component, with a content of 90% or more and a porous structure that absorbs water during mixing, thereby reducing fluidity. The quality of RHA varies depending on the calcination environment; however, the effect is not consistent. In this study, the pore structure was modified, and fluidity was improved by adjusting the particle size of the RHA. From a quality control perspective, this study aims to classify grades using Luxan values. While the characterization of RHA is based on Luxan values, the methodology for measuring its hydration response has not been reviewed. The test methods used in this study are as follows. To test the raw materials, density, specific surface area, XRF, SEM, and isothermal adsorption–desorption curves were measured, and fluidity was measured in fresh mortar. In a hardened mortar, compressive strength and drying shrinkage length change rate were measured. In addition, XRD and TG were measured for specimens after the compressive strength test. The selective dissolution method was used to measure the hydration rate. By adjusting the particle size of RHA to 45 µm, fluidity was enhanced. The relationship between the Luxan value and the basic properties of the mortar indicates that higher Luxan values correspond to greater compressive strength and drying shrinkage. We believe that the method used in this experiment can be used to quantify RHA.

## 1. Introduction

Rice husks are an agricultural byproduct with an estimated annual discharge of 120 million tons globally [1]. The husks contain approximately 15–20% SiO_2_ by mass [2]. RHA (rice husk ash) has about 90% silica, so it is a pozzolanic reaction material such as fly ash and silica fume, which are conventionally used concrete admixtures. According to previous studies, RHA contains a large amount of silica and can induce a pozzolan reaction, such as fly ash and silica fume. In addition, in fire resistance tests, the fire resistance performance was higher than that of ordinary concrete [3,4,5,6,7,8,9]. As such, RHA is actively being reviewed for use in concrete to reduce the environmental load and recycle resources. However, processing methods for rice husks have not yet been established, and existing treatment methods are particularly challenging due to the substantial amount of amorphous silica they contain. Recent research has identified a method for producing RHA with a high content of amorphous silica by controlling the burning temperature and calcination process [10,11]. The RHA obtained via this method exhibits a SiO₂ content nearly equivalent to that of silica fume and is currently being investigated as a potential new pozzolanic material.

In addition, when RHA is substituted for cement in concrete, a positive effect is shown, and the effect according to the substitution rate is also considered. Regarding the use of RHA, the method of substituting for cement is generally performed, and different properties are shown depending on the substitution method. However, it is reported that the substitution amount of RHA is generally 10% to 20% [12,13,14,15,16,17,18,19,20,21]. The properties of RHA concrete have been reported to increase long-term compressive strength and decrease autogenous shrinkage and durability [22,23,24,25]. Breugel V. K. and Ye G. Tuan V. N. substituted 10% and 20% RHA and compared it with silica fume. When the particle size of RHA was smaller, the long-term compressive strength at 91 days was 13% higher, and the autogenous shrinkage was reduced by 90% [22]. Additionally, Van V. et al. compared silica fume with a 10% replacement of RHA and reported that the rate of change in self-shrinkage length was about half that of silica fume [24]. In addition, experiments to confirm the effect of adjusting the particle size of RHA have been reported, and silica fume exhibits comparable mechanical properties according to the adjustment of the particle size [26,27,28,29]. Adjusting the particle size showed an increase in compressive strength of nearly two times [26]. Xu W. et al. controlled the particle size to 7 μm, which showed a compressive strength twice as high as that of basic RHA [27]. In the study by Venkatanarayanan H. K. and Rangaraju P. R., the size of RHA was adjusted to less than 10 μm, and it showed a compressive strength 10% higher than that of RHA without particle size adjustment [29].

There are two main factors that affect the production quality of RHA as an admixture in concrete. First, temperature and time are essential factors in the calcination process. Andres Salas et al. reported the effect of the change in the calcination method. RHA, to which chemical and calcination treatment was applied, had more amorphous properties and showed positive results in increasing the compressive strength of concrete [30]. Lingling Hu et al. [31] performed hydrochloric acid treatment as a pretreatment and experimented with changing calcination temperatures. In this study, after pretreatment with hydrochloric acid, calcination at 600 °C was used and showed higher performance compared to other admixtures. Hossein Beidaghy Dizaji et al. [32] studied the effect of changes in the RHA pretreatment method on physical properties, and when the pretreatment method was applied, the specific surface area was higher. In addition, pretreatment in the calcination process showed a higher SiO_2_ production rate. Ayesha Siddik et al. [33] reported that producing RHA with more than 80% amorphous silica was possible when incinerated at 700 °C. In addition, it has been reported that the smaller the RHA particles, the more active the pozzolan reaction. RHA particles of 20 mm or less at 30% or less are reportedly optimal. Sung-Hoon Kang et al. [34] reported that calcination at 650 °C for 2 h is effective in producing RHA with an amorphous silica content of 90% or more. In addition, black RHA and white RHA were used in calcination, and white RHA positively affected compressive strength. White and black RHA come from different firing temperatures, where firing at 650 °C produces white RHA. Deepa G Nair et al. [35] studied amorphous silica according to calcination temperature. Amorphous silica with high RHA calcined at 500 °C and 700 °C was contained and showed good pozzolanic reaction. In addition, it was reported that crystals were formed at 900 °C. Jose James and M. Subba Rao [36] tested the reactivity of RHA according to the calcination temperature, and as a result, RHA calcined at around 500 °C had the highest reactivity. Alias Mohd Yusof et al. [37] studied the conditions for producing RHA. The calcination process and washing of RH were used as variables; the calcination in a plug flow reactor produced fewer impurities than calcination in open burning, and the washing process with water resulted in fewer impurities. Ru-Shan Bie et al. [11] investigated the effect of changes in calcination temperature and calcination time on compressive strength when RHA is produced. It has been reported that the lower the firing temperature and the longer the firing temperature, the more positive the compressive strength. M.F.M. Zain et al. [38] studied the calcination method of RHA and reported that high-quality RHA could be obtained by supplying sufficient air.

Conversely, no straightforward approach exists to quantify the activity of RHA as a pozzolanic material, aside from measurement methods employing X-ray diffraction (XRD) or X-ray fluorescence spectrometry (XRF). Consequently, the current situation presents a challenging context for determining RHA quality. Luxán presented a method for measuring the reaction of pozzolanic materials [39] to gauge material performance [11,40].

RHA is reported to have pores as small as 10 µm [11], which can adversely affect compressive strength and fluidity when it is used as a replacement in concrete [41]. While the use of fly ash as a pozzolanic material is widespread, with research focusing on its particle size and fluidity [42], similar research on RHA in this context has not been conducted. Additionally, C-S-H content reportedly increases when RHA is partially replaced with cement [43]. Although many studies have focused on RHA, most of the literature is concerned with its preprocessing [31,32,44,45,46]. No methods have been proposed to improve fluidity other than using superplasticizers (SP) or increasing the water content in the formulation. Moreover, studies on the reaction ratio and measurement method for RHA remain unclear [47].

A synthesis of RHA studies reveals three key areas requiring further investigation: the absence of a reliable method for measuring the pozzolanic reaction of RHA, the need to address fluidity issues associated with RHA, and the necessity of developing a robust approach for quantifying RHA quality. Accordingly, we have focused our efforts on developing solutions to these problems.

The objective of this study is to investigate the potential for increasing the use of RHA in concrete. To address the poor fluidity of RHA, which has been identified as a disadvantage under certain conditions, RHA particle size is adjusted to determine the effects of this adjustment. Additionally, this study aims to clarify the relationship between RHA quality and Luxan values. Furthermore, a method for measuring the hydration rate of RHA is presented.

## 2. Materials and Methods

### 2.1. Specimen Overview

**Figure 1:**
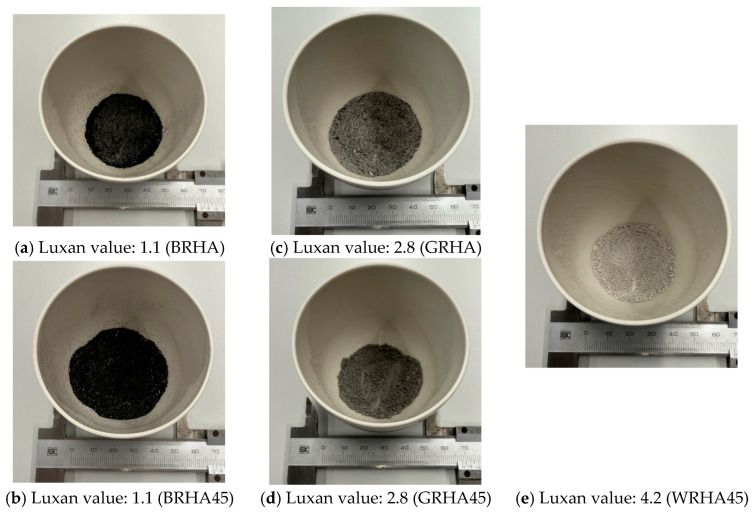
RHA according to the change in calcination conditions.

In this study, three types of RHA with Luxan values of 1.1, 2.8, and 4.2 were used. The grades were chosen as 2.8 for universally produced products, 1.1 for low-quality products, and 4.2 for high-quality products. Two types of particle sizes—less than 45 µm and fine pulverization—were employed. The test specimens were fabricated using a steel mold with dimensions of 40 × 40 × 160 mm. Six levels were prepared: one mix with varying levels of RHA and another mix with only ordinary Portland cement (OPC). The mixture compositions for each level are presented in Table 1. The RHA replacement rate for cement was set at 15%. Figure 1 shows the three types of RHA used, black, gray, and white, with Luxan values of 1.1, 2.8, and 4.2, referred to as B, G, and W, respectively. The properties of the materials used in the study are listed in Table 2. Mixing, curing methods, and compressive strength measurements were performed in accordance with JIS R 5201 [48]. Cement and RHA were thoroughly mixed, and water was added. After 24 h, the specimens were demolded and cured in water. Compressive strength tests were conducted at various ages. The dry shrinkage test was performed one week after demolding, with measurements taken in a controlled climate of 20 °C and 60% humidity.

Table 3 shows the basic properties of OPC. Table 4 lists the details of the paste specimens used in the hydration rate experiments. Three types of main particles were used, and the Luxan value was tested for two particle sizes. The RHA replacement rate was set at 15%, and the W/C was set to 50%.

### 2.2. Particle Size Control

**Figure 2:**
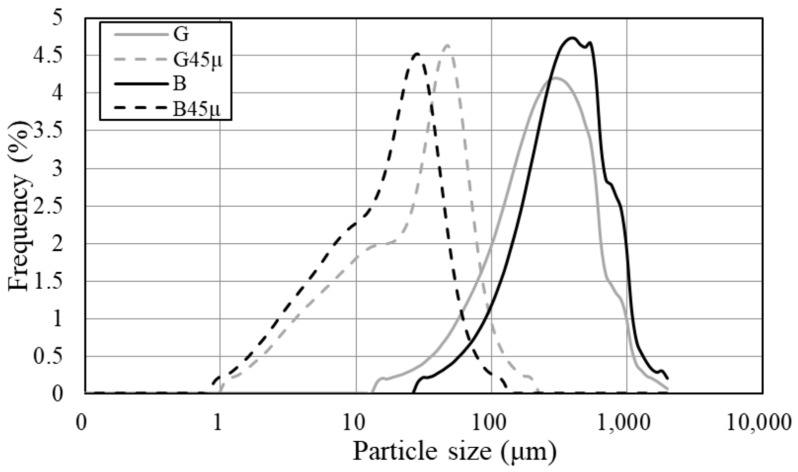
Particle size distribution.

**Figure 3:**
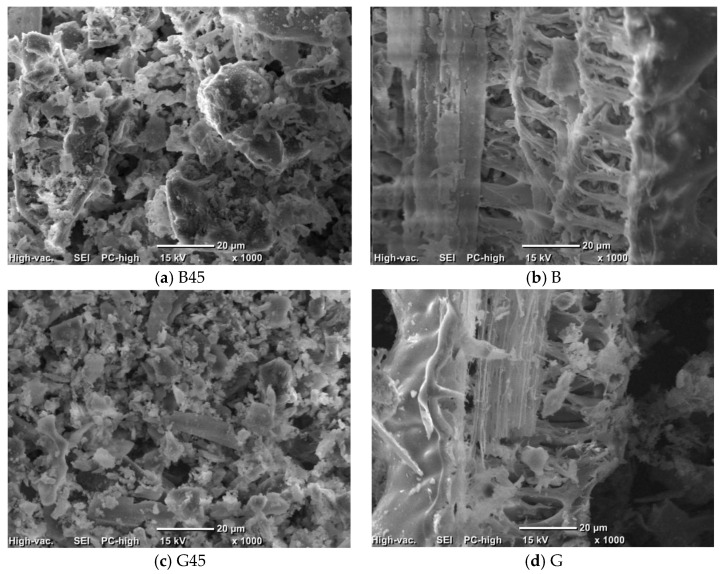
Scanning electron microscopy (SEM) of RHA.

A ball mill was used to control the particle size of the RHA. Two ceramic balls with diameters of 10 mm and 3 mm were used, and both the mill and the balls were made of ceramic. To achieve a particle size of 45 microns, the mill was set to a spinning speed of 600 rpm for 60 min. The particle size distribution curve obtained after particle size adjustment is shown in Figure 2. The experiment was performed in accordance with JIS R 5201 [48]. The particle size was measured using a laser diffraction/scattering particle size analyzer (LA-950V2, Kyoto, Japan), with the main particle size of the raw material being 500 μm and the adjusted material being 45 μm. Figure 3 shows the scanning electron microscopy (SEM, JEOL/JCM-6000, JEOL Ltd., Tokyo, Japan) image of the raw material and the resulting image after particle size control. It is evident that the voids present in the RHA particles are eliminated after particle size control. As shown in Table 5, the results of the X-ray fluorescence (XRF, Rigaku/ZSX) analysis indicate that particle size control had no effect on mineral fracture.

### 2.3. Test of Compressive Strength

A compressive strength test was performed in accordance with JIS R 5201 [48]. The compressive strength was measured at 7, 28, and 91 d, and the average of three tests at each age was taken as the final compressive strength, calculated using Equation (1). The loading speed was set at a rate of 50 ± 10 N per second to obtain the maximum load. The specimen used for the compressive strength test was a 40 × 40 × 160 mm sample, which was divided into three pieces of 40 × 40 × 40 mm for each specimen.

(1) C=W/1600

where *C*: Compressive strength (N/mm^2^), and *W*: Maximum load (N).

### 2.4. Test of Drying Shrinkage

Drying shrinkage was determined by curing the specimens under water for 7 d after pouring the mortar, with the initial point of drying starting after 7 d. The drying shrinkage was measured for 91 d using an embedded gauge. This experiment was also performed in accordance with JIS R 5201 [48].

### 2.5. Test for Density

Density was tested following the JIS R 5201 standard [48]. Measurements were conducted using a Le Chatelier flask and kerosene. The sample used for density measurement had an unadjusted RHA of 45 microns.

### 2.6. Water Vapor Isothermal Adsorption Test

A water vapor isothermal adsorption test was conducted to evaluate the changes in pore structure after particle size control. An isothermal adsorption curve was obtained using a water vapor adsorption method with an equilibration time of 60 s. Measurements were taken at increments of 5% relative humidity (RH) in the range of 5–98%, with the maximum value of 98% RH being repeated. The specific surface area resulting from water vapor adsorption was calculated using the Brunauer–Emmett–Teller theory based on data derived from the desorption process within the RH range of 10–35% [49]. In this method, the area occupied by water was determined to be 0.125 nm.

### 2.7. Thermogravimetric Differential Thermal Analysis (TG-DTA)

To determine the quantity of calcium hydroxide generated through the hydration of cement in the pozzolanic reaction with RHA, Joule differential thermal analysis was conducted. The temperature range was set between 30 and 1000 °C, with a heating rate of 20 °C per minute. A TG/DTA 7200, manufactured by Hitachi (Tokyo, Japan), was used for the analysis, with samples adjusted to under 300 µm. α-Alumina was used as the reference sample, and the sample weight was set to 20 mg. Additionally, 120 mL/min of nitrogen gas was utilized for TG.

CH and CC were calculated according to Equations (2) and (3) below.

Calcium hydroxide generally dehydrates (decomposes into CaO and H_2_O) at around 400–500 °C. The weight change is denoted as *WL_Ca(OH)*2*_*, and the molecular weights of calcium hydroxide, *m_Ca(OH)__*2*_* = 74, and that of water, *m_(H__*2*__O)_* = 18, are used to determine the amount of calcium hydroxide.

(2) $${Ca\left(OH\right)}_{2,measured}={WL}_{{Ca\left(OH\right)}_{2}}\times \frac{m_{{Ca\left(OH\right)}_{2}}}{m_{\left(H_{2}O\right)}}={WL}_{{Ca\left(OH\right)}_{2}}\times \frac{74}{18}$$

Calcium carbonate is generally decarburized (decomposed into CaO and CO_2_) at about 600–700 °C. The weight change is determined as *WL_CaCO__*3*_*, and calcium hydroxide is determined using the molecular weight of calcium carbonate, *m_CaCO__*3*_* = 100 and CO_2_, *m_CO__*2*_* = 44.

(3) $${CaCO}_{3,measured}={WL}_{{CaCO}_{3}}\times \frac{m_{{CaCO}_{3}}}{m_{CO_{2}}}={WL}_{{CaCO}_{3}}\times \frac{100}{44}$$

### 2.8. X-Ray Diffraction (XRD) Analysis

For XRD measurements, the samples collected from each curing age were pulverized to a particle size of less than 300 µm. The measurement conditions included a voltage of 30 kV, a tube current of 10 mA, a step width of 0.01°, and a diffraction angle range of 2θ = 5° to 75°. Additionally, 10% α-alumina was mixed with the samples for analysis using the Rietveld method. TOPAS V5 (manufactured by Bruker AXS, Billerica, MA, USA) was used for Rietveld analysis, and the data were interpreted based on the XRD measurements. The amount of amorphous content was determined by comparing the amount of calcium hydroxide in TG-DTA with the amount of reference material (α-alumina).

### 2.9. Methodology for the Quantification of RHA

The selective dissolution method was employed to quantify the hydration rate of RHA, following a methodology previously used for quantifying fly ash [14]. The powdered sample was treated with a solution containing 2N hydrochloric acid and 5% sodium carbonate to dissolve cement and cement hydrates. The mass of each sample was measured after drying.

### 2.10. Determination of Unreacted RHA from Hardened Cement Paste

Table 6 presents the results of the hydration-rate experiments. Reaction rate tests were conducted using the selective dissolution method proposed by Osawa et al., which effectively recovered unreacted fly ash [47]. Since RHA primarily consists of SiO₂ and exhibits properties similar to fly ash, the quantity of residual RHA was determined using a comparable method. A solution of 2N hydrochloric acid and 5% aqueous sodium carbonate was added to the powder sample to dissolve the cement and cement hydrates. The mass of each sample was then measured after drying. The insoluble residual rates for each sample were 93.07% and 85.44%, respectively. Reaction rates were corrected using the calculated insoluble residual rates. The specimens were cured in water, and measurements were taken at each curing age.

### 2.11. Calculation of Luxan

The Luxan value, proposed by Luxan, is a simple method for measuring the pozzolanic reaction and is known to be effective in measuring the amount of amorphous silica [39,40]. Generally, a higher Luxan value indicates a higher active value.

After determining the following two measurements, the result is derived by substituting it in Equation (4).

(1) The electrical conductivity is measured before adding 40 mL of calcium hydroxide (value a; mS/cm).
(2) RHA sample (1.0 g) is added to the calcium hydroxide mixture, and the electrical conductivity is measured after 2 min (value b; mS/cm).

Luxan = value a − value b(4)

For the preparation of the saturated calcium hydroxide solution used in this study, 0.64 g of calcium hydroxide reagent was added to 500 mL of pure water and mixed in an environment of 40 °C.

## 3. Results

This section is divided into three subsections. First, basic materials are reviewed to verify the impact of particle size adjustments. Second, the fundamental characteristics of the mortar, including flow, compressive strength, and drying shrinkage, are examined. Finally, investigations involving the assessment of methodologies used for quantifying the RHA hydration rate are discussed. The results of the fundamental characteristics of the mortar experiments represent the average of three specimens.

### 3.1. Basic Materials

#### 3.1.1. Density

**Figure 4:**
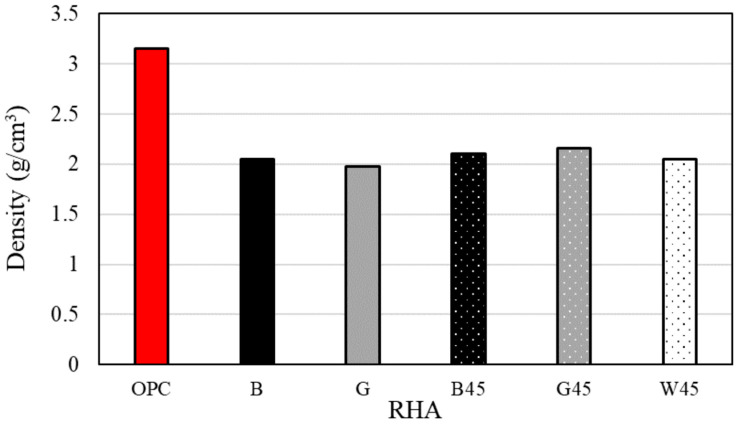
Density according to RHA.

Figure 4 presents the data obtained from the density measurements of various powder materials. This study hypothesized that the pores in RHA would be destroyed during particle size adjustment. However, the density measurement results indicated that the density of RHA at 45 microns exhibited only a slight increase, suggesting that an increase in weight per unit volume contributed to the observed effect.

Previous studies have indicated that an increase in the substitution rate of RHA is associated with a reduction in density, which is attributable to the presence of pores in RHA [4,16,24,50]. Additionally, the density of secondary pores (0.1–100 μm) increases with the substitution rate of RHA, and the density of these secondary pores is sensitive to changes in the proportion of RHA [12]. Further research has also shown that the pore structure of RHA reduces the density of materials [22].

#### 3.1.2. Water Vapor Isothermal Adsorption Test Results

**Figure 5:**
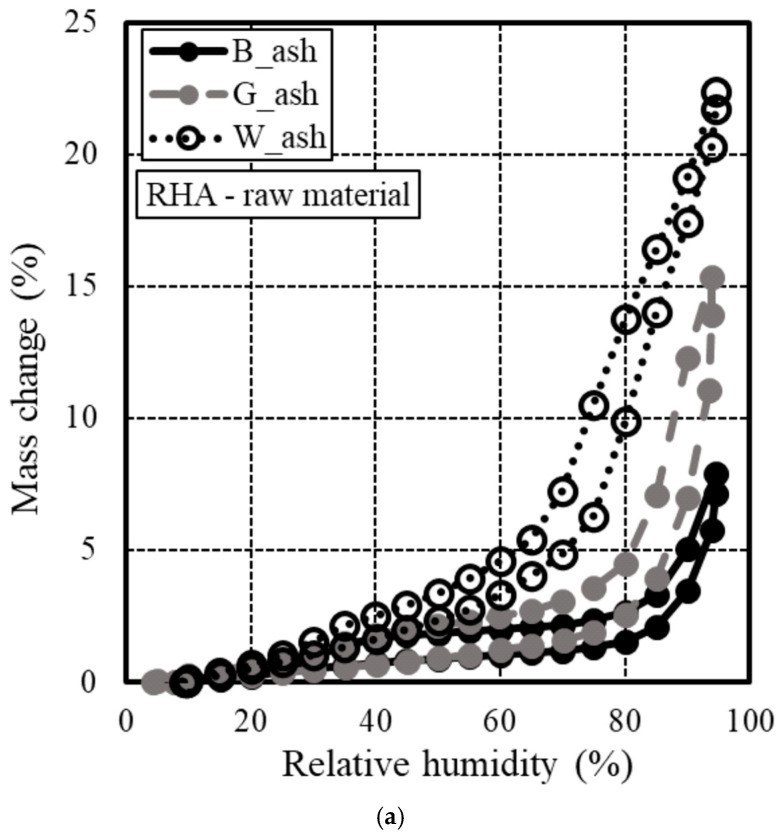

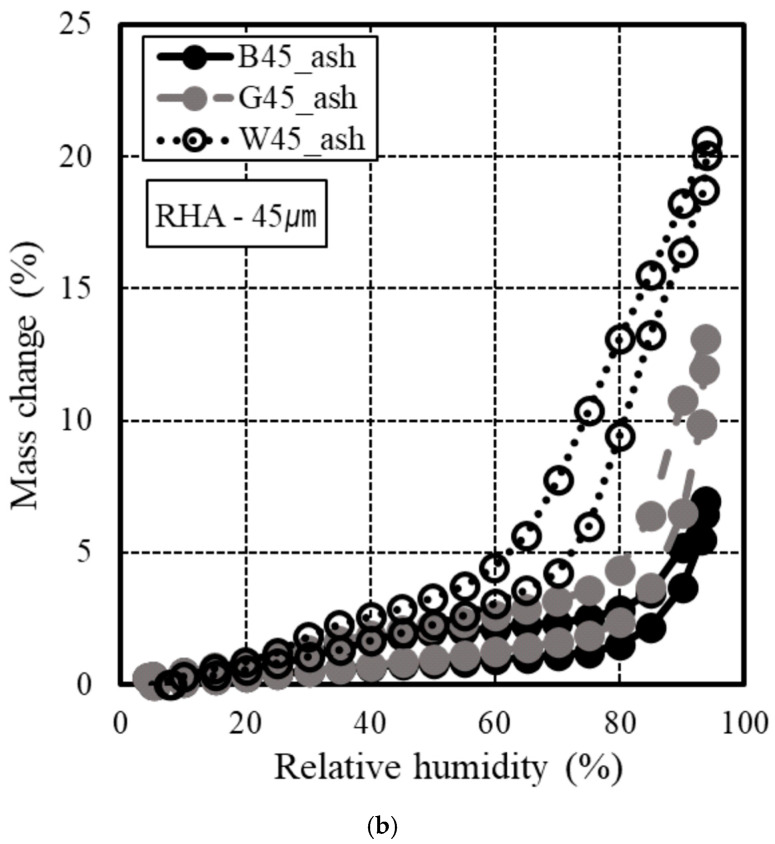
Isothermal adsorption–desorption curve for RHA. (**a**) raw material, (**b**) 45 μm.

As shown in Figure 5, the isothermal adsorption–desorption curve yielded the following results: specimens with elevated Luxan values exhibited a notable increase in weight due to water vapor adsorption, indicating the presence of a substantial quantity of mesopores. Additionally, the RHA sample with a 45 μm particle size adjustment demonstrated comparable outcomes to the raw RHA in terms of color. In terms of weight change, the 45 μm particle size-adjusted specimen had a lower weight compared to the raw RHA material, suggesting that the microstructure of the RHA was affected. This outcome is attributed to the influence of the porous RHA structure, which operated in a manner similar to that previously described [12,22].

**Figure 6:**
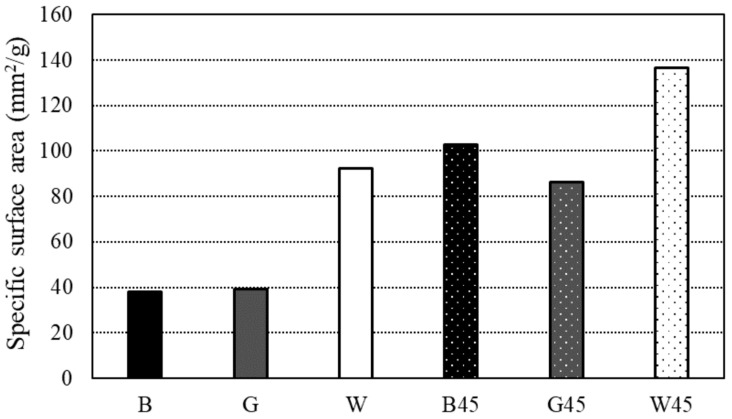
Specific surface area by BET.

Figure 6 shows the results for specific surface areas. The specific surface area, calculated using the BET method from the isothermal adsorption/desorption curves in the RH range of 10~35% during desorption, revealed that the two raw material samples, B and G, exhibited nearly identical results, while W had a larger specific surface area compared to the others. Additionally, the particle size-adjusted samples generally had a higher specific surface area than the raw material samples, with B45 and G45 showing results similar to those of W, and W45 exhibiting a more pronounced increase.

### 3.2. Fundamental Characteristics of the Mortar

#### 3.2.1. Flow Test

**Figure 7:**
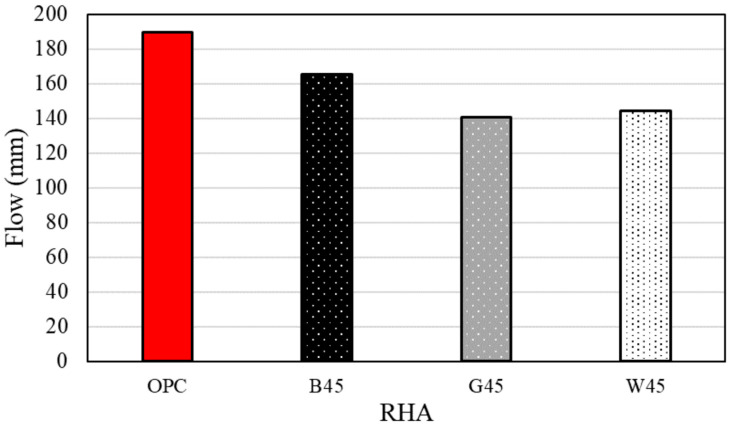
Flow according to RHA replacement.

**Figure 8:**
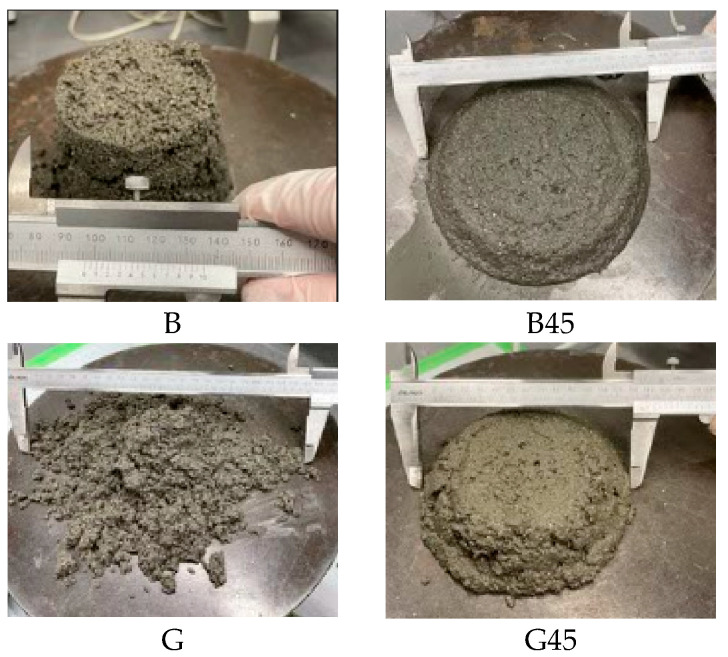

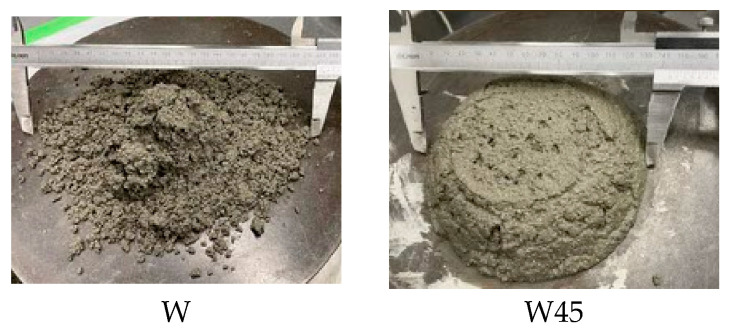
Flow test photographs according to RHA replacement.

Figure 7 and Figure 8 present the results and a photograph of the flow experiment, respectively. As shown in Figure 8, flow measurements could not be conducted on the test specimen with RHA before particle size adjustment due to moisture absorption. The specimens containing 45 µm RHA exhibited reduced absorption of mixing water, resulting in improved flow. B, with a low Luxan value, demonstrated a high flow value, which inversely correlated with the maximum weight change observed in the isothermal adsorption curve (Figure 5). This phenomenon is attributed to the reduction in RHA micropores identified during grain-size adjustment [11], which positively influenced fluidity. Additionally, other studies have reported that the pore structure of RHA absorbs water, a finding confirmed in this study [22,24,47].

Previous literature has used RHA with particle sizes up to 75 μm, which often led to increased formulation complexities. However, the present study used RHA with particle sizes of 46 μm or less and demonstrated improved fluidity [31]. Additionally, as shown in Figure 3, the particle size control ruptured the RHA pores.

#### 3.2.2. Compressive Strength TEST

**Figure 9:**
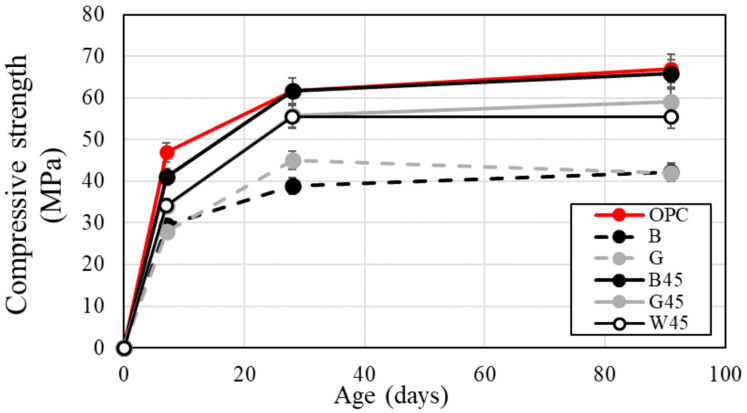
Compressive strength according to RHA replacement.

Figure 9 illustrates the variation in compressive strength with respect to RHA substitution. Overall, specimens containing RHA with adjusted particle sizes exhibited higher compressive strength than those with raw RHA. By adjusting the RHA particles, high compressive strength was achieved at all ages. In addition, the samples before particle control showed no difference between B and G. However, after particle control, B showed higher compressive strength.

This trend is attributed to the increased specific surface area resulting from particle size adjustment. Comparative analysis of RHA specimens B45, G45, and W45 revealed that B45 consistently demonstrated the highest compressive strength at all curing ages, outperforming the other two specimens. Additionally, RHA with adjusted particle sizes showed a tendency for enhanced compressive strength, likely due to improved bonding with aggregates [26,51]. The compressive strengths of specimens G45 and W45 were similar. Slope analysis of the graph in Figure 9 showed that the RHA specimens with adjusted particle sizes exhibited a significantly greater increase in compressive strength from 7 to 28 d compared to the OPC. However, the increase in compressive strength from 28 to 91 d was not significantly different from that observed for the OPC. These findings suggest that the pozzolanic reaction was most active between 7 and 28 d. The rate of increase in compressive strength was a function of curing age. Reducing the RHA particle size led to a higher rate of increase in compressive strength from 7 to 28 d. The data indicate that finer RHA particles resulted in a greater increase in compressive strength during this period. Furthermore, the relationship between the Luxan value and compressive strength suggests that the rate of strength increase correlates with the Luxan value, indicating that the Luxan value is proportional to the pozzolanic reaction of amorphous silica. The increase in compressive strength due to RHA is attributed to both the filler effect and pozzolanic reaction [52]. At 28 d of curing, the compressive strength of specimens substituted with raw RHA decreased, consistent with findings from other studies. This decrease is believed to result from an increased number of voids associated with RHA use [4,10,16].

Additionally, it has been reported that the rate of increase in compressive strength at long-term curing ages is higher than at initial curing ages, which is consistent with findings from previous studies [14,19,28,53]. It was also noted that the number of voids and water absorption decreased inversely with compressive strength [53].

Breugel et al. [22] reported that the curing reaction occurs within the pores of RHA, suggesting that the reaction is a complex process involving both pozzolanic and curing reactions within the RHA voids. Zerbino et al. [26] and Giaccio et al. [51] described the characteristics of RHA with and without particle size adjustment, noting that strong bonding to the aggregate and cement matrix was achieved when the RHA was substituted in appropriate amounts. Therefore, it is concluded that adjusting the particle size appropriately can yield a similar performance to OPC using only cement.

#### 3.2.3. Dry Shrinkage Test

**Figure 10:**
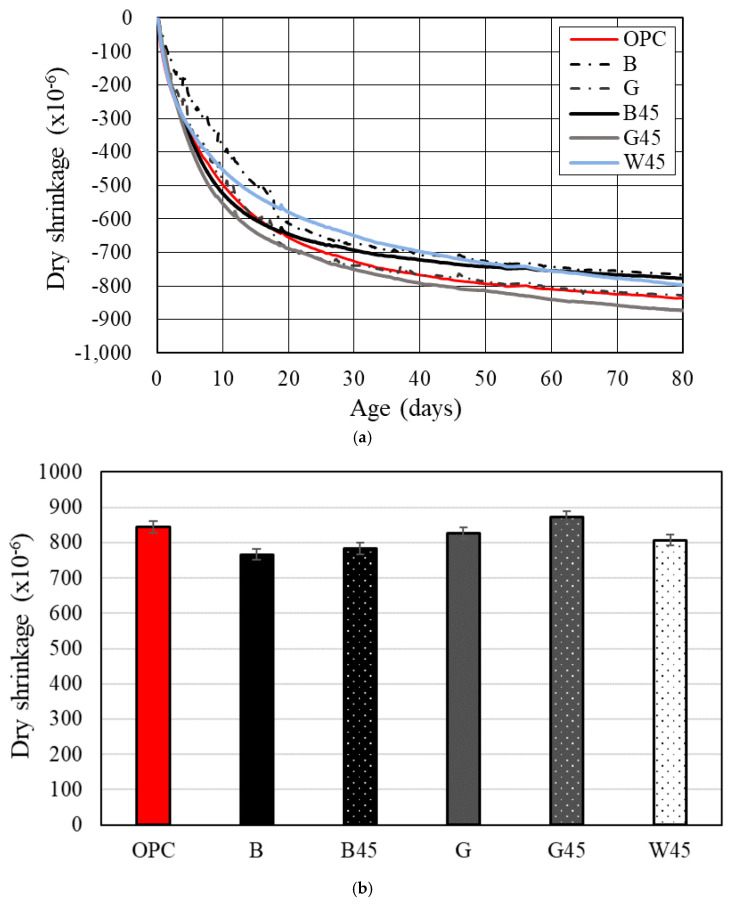
Dry shrinkage according to RHA replacement. (**a**) dry shrinkage; (**b**) drying shrinkage strain.

Figure 10a displays the dry shrinkage results for each specimen based on the average data obtained from embedded gauges on three specimens. Except for G45, the RHA specimens exhibited dry shrinkage results that were either similar to or lower than those of the OPC. The RHA test specimens with particle control showed lower drying shrinkage than the test specimens without particle control. In addition, B showed lower drying shrinkage overall than G. A comparison of the dry shrinkage strain between raw RHA and 45 µm RHA specimens revealed that the latter had a greater tendency towards larger dry shrinkage. B45 showed compressive strength comparable to OPC but with reduced drying shrinkage relative to OPC. Figure 10b illustrates the drying shrinkage strain at 80 d. The results for RHA specimens B, G, B45, and G45 indicated that a lower Luxan value corresponded to a smaller dry shrinkage strain. This observation aligns with the trends reported in the literature [12,14].

In a previous study, RHA substituted for calcined dolomite resulted in decreased dry shrinkage as RHA content increased [14]. The dry shrinkage results in this study were similar to those of the plain specimens. Similarly, Wang et al. [12] reported that pore volume, which decreased with curing age, tended to increase with a higher RHA substitution ratio. This suggests that the hydration products generated by the pozzolanic reaction may fill the pores, explaining the observed increase in compressive strength with curing age compared to the plain specimens.

In the autogenous shrinkage experiment by Breugel et al. [22], it was reported that RHA with a particle size of 5.6 µm or larger reduces autogenous shrinkage because the curing reaction occurs within the pores of the RHA. Therefore, proper particle size control is expected to positively impact drying shrinkage.

Additionally, studies by Viet-Thien-An Van et al. [24] and Gemma Rodriguez de Sensale et al. [54] reported that RHA substitution contributed to a reduction in autogenous shrinkage. Similarly, Hu et al. [31] found that the creep rate decreased with an increased substitution rate of RHA. Although the experimental conditions varied, the results suggest that reducing capillary voids had a beneficial effect, with RHA substitution showing improvements when the particle size was greater than 10 µm.

#### 3.2.4. Thermogravimetric/Differential Thermal Analysis (TG-DTA)

**Figure 11:**
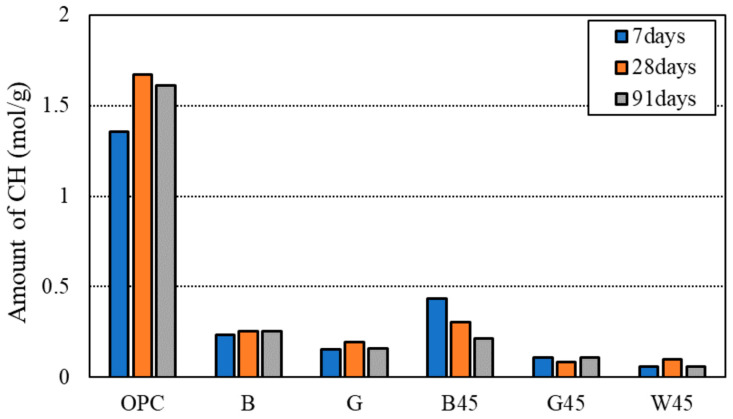
Amount of calcium hydroxide according to RHA replacement.

Figure 11 displays the calculation results for the amount of Ca(OH)_2_ produced, as determined by TG-DTA. The quantity of Ca(OH)_2_ was calculated based on the mass loss observed between 350 and 450 °C on the thermogravimetric (TG) curve. The results indicated that RHA substitutions led to specimens with lower amounts of calcium hydroxide compared to those with OPC. This reduction is attributed to the consumption of calcium hydroxide during the pozzolanic reaction. Furthermore, a comparison of raw RHA and 45 μm RHA specimens showed that, except for B45, the 45 µm RHA specimens had lower concentrations of calcium hydroxide. Despite this, the B45 specimen exhibited the highest compressive strength among the RHA-substituted specimens, suggesting the need for further investigation into the relationship between the RHA pozzolanic reaction and compressive strength. The data indicated that calcium hydroxide consumption through the pozzolanic reaction was consistent with trends observed in previous studies [8,21,47,54,55].

Additionally, it has been reported that a reduction in calcium hydroxide and a decrease in pores in the interfacial zone are associated with improved physical properties [21]. In an experiment by Rêgos et al. [23], varying the amount of amorphous silica with a 20% RHA substitution showed that a higher amount of amorphous silica increased the pozzolanic reaction due to greater consumption of calcium hydroxide. These findings correlate with the Luxan value used in this study, reflecting similar trends.

Tuan et al. [8] reported that water absorbed by the porous structure of RHA can enhance the hydration reaction of cement, leading to improved strength development. Additionally, both calcium hydroxide and RHA are known to undergo a pozzolanic reaction. This effect is believed to contribute to the simultaneous decrease in calcium hydroxide and increase in compressive strength observed in this study.

In cases where RHA had a high Luxan value, more calcium hydroxide was consumed, and higher compressive strength was achieved at later ages. This suggests that the Luxan value is proportionally related to the amount of amorphous silica. Conversely, in the mechanism of strength development by RHA, it was observed that the water absorbed by RHA mesopores, rather than the consumption of calcium hydroxide, lowered the W/B ratio of the mixture. This reduction in W/B ratio is believed to positively influence the pozzolanic response over long curing periods, as reported by Viet-Thien-An Van et al. [24].

#### 3.2.5. X-Ray Diffraction (XRD)

The objective of this study was to determine whether the particle size of RHA influenced the minerals produced during the hydration reaction and to assess whether the pozzolanic reaction led to the consumption of CH.

**Figure 12:**
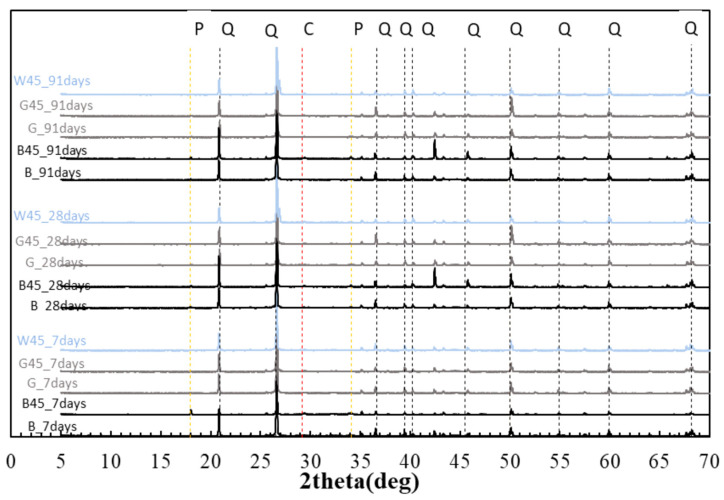
XRD of RHA (P: portlandite, C: calcite, and Q: quartz.

Figure 12 presents the XRD measurement results for specimens with RHA replacement. Quartz was consistently detected at all levels and ages, as the samples were obtained from mortar test specimens. Additionally, the portlandite peak near 18° on the horizontal axis confirmed the presence of portlandite in the B45 specimens at each age. Although a minimal peak was observed at 91 d in other specimens, these results were largely consistent with the calcium hydroxide quantities derived from the TG-DTA results.

**Figure 13:**
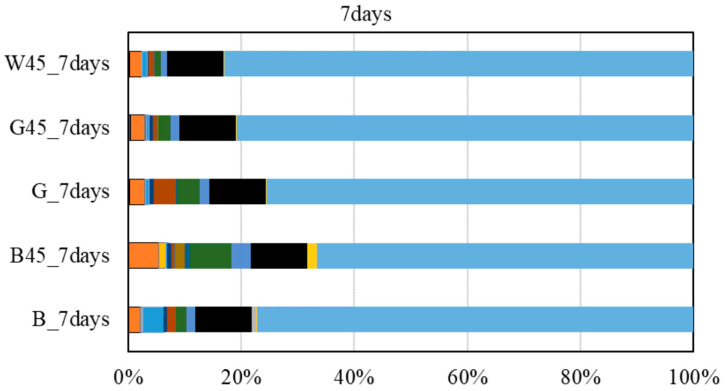

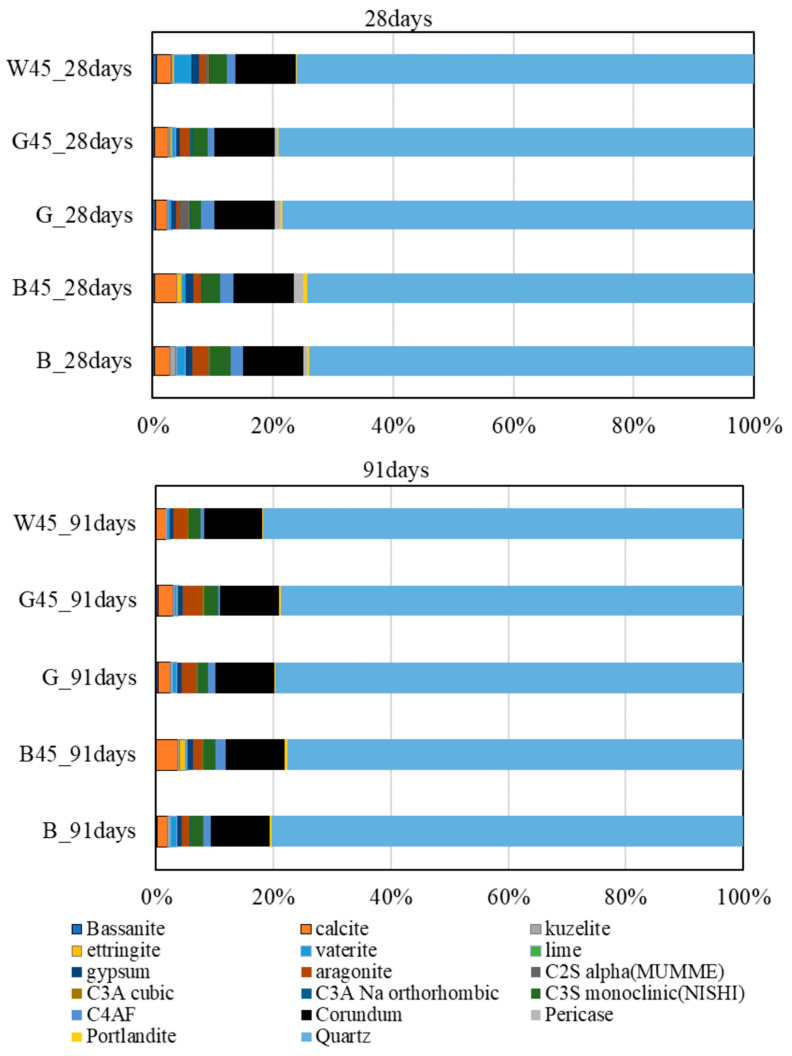
Rietveld method for RHA.

Figure 13 displays the results of the Rietveld method for quantification. The quantity of quartz remained consistent, with the exception of the results for the 7 d curing age.

#### 3.2.6. Results of the RHA Reaction Rate Test

**Figure 14:**
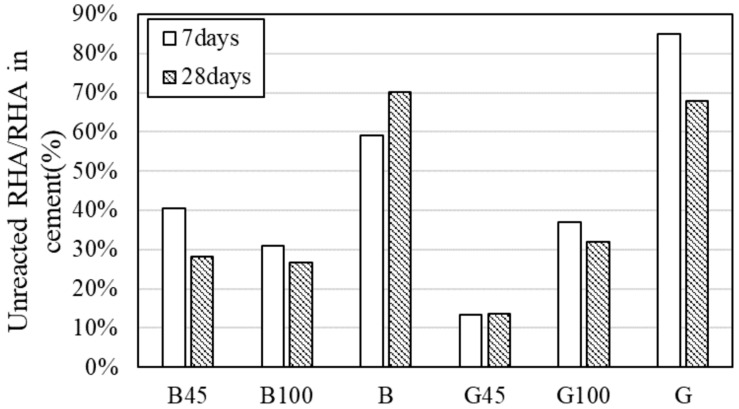
RHA reaction rate test results using the selective dissolution method.

Figure 14 presents the RHA reaction rate test results using the selective dissolution method. For all test specimens, excluding the raw material and G45, the amount of unreacted RHA tended to decrease as the curing age increased. This indicates that the hydration reaction of RHA progressed from 7 to 28 d. Comparing the raw material and G, the difference in reaction rate at 28 d was only about 4%, with values at 7 d being similar to those of uncrushed G. For G45, approximately 85% had reacted by 7 d, indicating improved reactivity of RHA due to particle size adjustment.

Regarding the Luxan value, G exhibited a higher reaction rate than B in the 45 μm specimen. However, B reacted more than G in the raw material and 100 μm samples. Above 100 μm, the difference in reaction rate was approximately 10%, suggesting that the reaction rate could vary with curing age. Therefore, further investigations are warranted.

In terms of particle size, the difference in reaction rate between raw and crushed RHA was significant. Particle size adjustment notably affected the reaction rate even at early curing ages. Smaller particle sizes of RHA, which increase the specific surface area, promoted the hydration reaction. Thus, adjusting the particle size improved the reactivity of RHA.

#### 3.2.7. Calcium Hydroxide Determination

**Figure 15:**
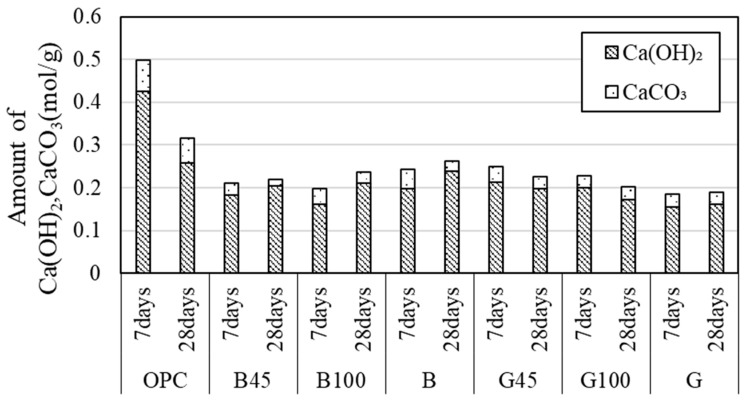
Quantitative results for calcium hydroxide and calcium carbonate.

Figure 15 presents the quantitative results for calcium hydroxide and calcium carbonate calculated from the TG-DTA measurement data. For B, the calcium hydroxide content tended to increase with material age. Since B had a low Luxan value, the production of calcium hydroxide through hydration reactions surpassed its consumption through the pozzolanic reaction. In contrast, no increase in calcium hydroxide was observed in G, suggesting that the pozzolanic reaction in G was more advanced than in BRHA. However, the differences in the quantitative results for the hydrates were minimal. Future investigations should focus on the quantitative amounts and reaction rates of RHA at extended curing periods when the pozzolanic reaction becomes more active.

## 4. Discussion

### 4.1. Effect of RHA Particle Size on Mortar

**Figure 16:**
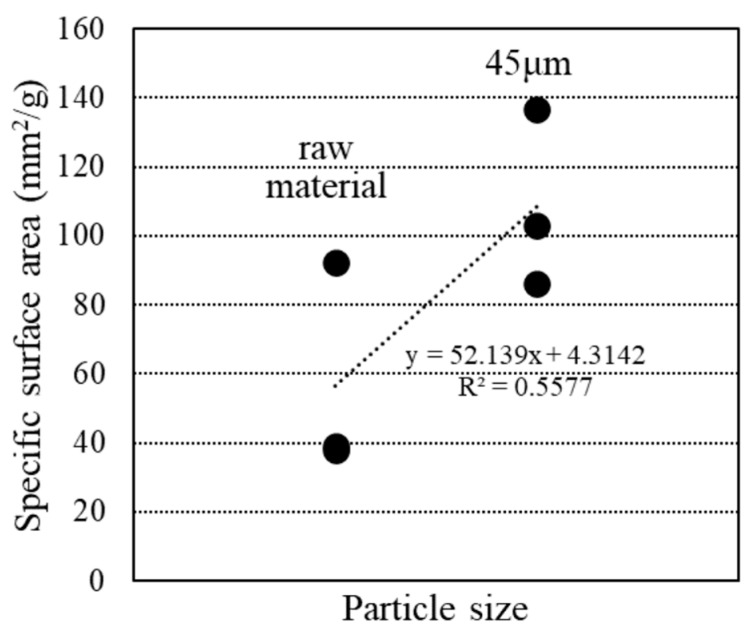
Relationship between particle size and specific surface area.

**Figure 17:**
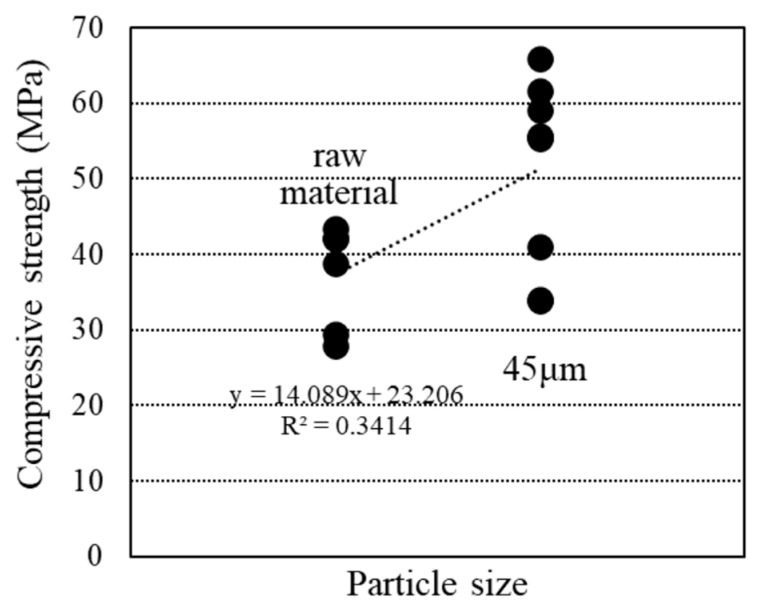
Relationship between particle size and compressive strength.

Figure 16 illustrates the relationship between specific surface area and particle size calculated using BET theory from the water vapor adsorption test data. The specific surface area increased when RHA was powdered. Adjusting the particle size of RHA disrupted its pore structure and improved flowability. Additionally, Figure 17 demonstrates a positive correlation between compressive strength and particle size. Reducing the particle size increased the specific surface area of RHA, which, in turn, contributed to enhanced compressive strength. Another study by Xu et al. [41] found that increasing the specific surface area of RHA improved its mechanical properties and degree of hydration.

### 4.2. Effect of Luxan Value on Mortar

**Figure 18:**
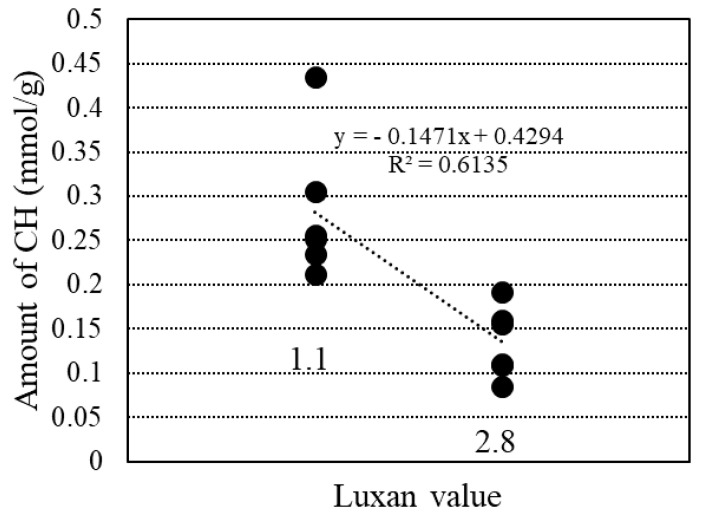
Relationship between CH amount and Luxan value.

Figure 18 shows the relationship between the amount of calcium hydroxide produced and the Luxan value, as calculated from the TG curve obtained from the TG-DTA data. The amount of calcium hydroxide produced decreased as the Luxan value increased, which was attributed to the pozzolanic reaction between the amorphous silica in the RHA.

**Figure 19:**
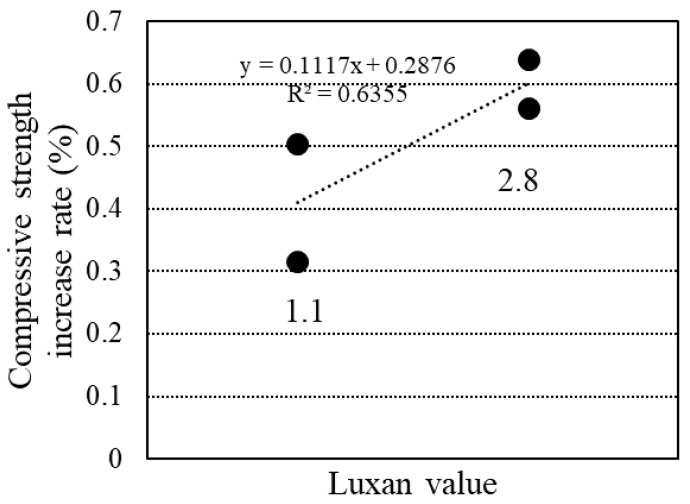
Relationship between compressive strength and Luxan value.

Figure 19 illustrates the relationship between the Luxan value and the rate of increase in compressive strength at 28 d compared to 7 d. A higher Luxan value was associated with a greater rate of increase, indicating that an increase in the Luxan value accelerated the pozzolanic reaction and resulted in a significant rise in compressive strength.

**Figure 20:**
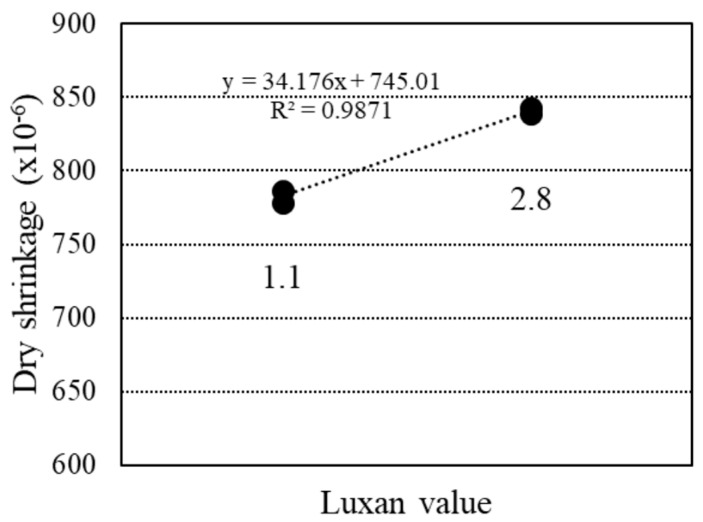
Relationship between dry shrinkage and Luxan value.

Figure 20 reveals a slight correlation between dry shrinkage at 80 d and the Luxan value. According to a study by He et al. [20], a higher RHA ratio generally reduced dry shrinkage strain; however, an upper limit effect was observed, with no significant difference in dry shrinkage strain above a certain RHA amount. Additionally, lower Luxan values had a minimal effect on reducing dry shrinkage at the same RHA ratio, although this effect was smaller compared to the influence of the RHA ratio used in this study [8].

With the exception of the relationship between Luxan value and dry shrinkage, the correlations were relatively weak. This may be due to the limited amount of data, indicating the need for additional data collection.

## 5. Conclusions

This study aimed to identify an effective method for processing RHA. To achieve this, it was necessary to determine the Luxan value for powdered RHA and adjust the particle size to address limitations associated with RHA, such as reduced compressive strength and increased particle size at early curing ages. Additionally, we demonstrated the feasibility of quantifying the hydration rate. The following conclusions were obtained:

(1) Compressive Strength: Adjusting the particle size leads to an increase in compressive strength proportional to the reduction in particle size. In addition, it showed a tendency to increase in proportion to the specific surface area.
(2) Effect of particle size control: Compressive strength increased 36% for B and 28% for G with particle size control. Dry shrinkage decreased 3.8% with particle size control and 4.5% without particle size control.
(3) Flow: Reducing the grain size of RHA improves the flow value, and the flow rate exceeds that of specimens without particle size adjustments. Adjusting the particle size effectively resolves fluidity issues associated with RHA mortar.
(4) High Luxan values: RHA with a high Luxan value, indicating elevated pozzolanic reactivity, is more effective in increasing compressive strength.
(5) Low Luxan value: RHA with a low Luxan value decreases the degree of drying shrinkage.
(6) The reaction rate varies significantly between raw RHA and powdered RHA, indicating that the powdering process substantially affects the reactivity of RHA, even during the initial curing period. We propose that adjusting the particle size of RHA can enhance its reactivity.

In future research, we aim to investigate whether the pH of recycled aggregate can be used to activate the pozzolanic reaction of RHA and contribute to the development of environmentally friendly concrete.

## Figures and Tables

**Table 1:** Mixture design of mortar specimen.

Sample	RHA		W/C (%) ^1^	Unit Weight (g) ^2^			
	Luxan Value	Main Particle Size (µm)		W	S	OPC	RHA
OPC	-	-	50	225	1350	450.0	-
B45	1.1	~45				382.5	67.5
B		-					
G45	2.8	~45					
G		-					
W45	4.2	~45					

^1^ W/C: water to cement ratio. ^2^ The unit weight used to make the 3 test pieces.

**Table 2:** Material characteristics of mortar specimen.

Materials	Symbol	Characteristics
Ordinary Portland Cement	OPC	Density: 3.15 g/cm^3^
Sand	S	Density: 1.29–1.31 g/cm^3^
Black RHA	B	Luxan Value: 1.1, Rapid Calcination at 600 °C, Particle Size: ~45 µm, Raw Material, Calcination: 180 min
Grey RHA	G	Luxan Value: 2.8, Rapid Calcination at 550 °C, Particle Size: ~45 µm, Raw Material, Calcination: 180 min
White RHA	W	Luxan Value: 4.2, Cooling After Calcination at 500 °C, Particle Size: ~45 µm, Raw Material, Calcination: 180 min

**Table 3:** Characteristics of OPC.

	Setting Time		Compressive Strength (MPa)			Chemical Composition				
	Initial Setting	Final Setting	3 d	7 d	28 d	MgO	SO_3_	Ignition Loss	Total Alkali	Insoluble Residue
OPC	2 h 30 min	4 h	30.4	48.3	67.0	0.92	1.98	2.39	0.57	0.021

**Table 4:** Mixture design of paste specimen.

Sample	RHA		W/C (%) ^1^	Unit Weight (g) ^2^		
	Luxan Value	Main Particle Size (µm)		W	OPC	RHA
OPC	-	-	50	225	450.0	-
B45	1.1	~45			382.5	67.5
B100		~100				
B		-				
G45	2.8	~45				
G100		~100				
G		-				

^1^ W/C: water to cement ratio. ^2^ The unit weight used to make the 3 test pieces.

**Table 5:** Measurement results of XRF of RHA (mass %).

Specimen	B	B45	G	G45
MgO	0.1	0.1	0.2	0.2
Al_2_O_3_	0.0	0.1	0.0	0.1
SiO_2_	92.0	92.7	90.4	91.6
P_2_O_5_	0.4	0.4	0.6	0.5
SO_3_	0.2	0.1	0.3	0.3
K_2_O	5.8	5.4	6.4	5.5
CaO	1.0	0.8	1.4	1.2
Cr_2_O_3_	0.0	0.0	0.0	0.0
MnO	0.3	0.2	0.3	0.3
Fe_2_O_3_	0.1	0.1	0.1	0.2
NiO	0.0	0.0	0.0	0.0
ZnO	0.0	0.0	0.0	0.0
SnO_2_	0.0	0.0	0.0	0.0
L.O.I.	0.9	2.3	1.4	2.5

**Table 6:** Measurement results of insoluble residual rate of RHA.

Specimen	Weight Before Dissolution (g)	Weight After Dissolution (g)	Weight After/Before Dissolution (%)
BRHA	1.01	0.94	93.07
GRHA	1.03	0.88	85.44

## Data Availability

The original contributions presented in the study are included in the article, further inquiries can be directed to the corresponding author.
